# MLKL post-translational modifications: road signs to infection, inflammation and unknown destinations

**DOI:** 10.1038/s41418-022-01061-5

**Published:** 2022-09-29

**Authors:** Gianmaria Liccardi, Alessandro Annibaldi

**Affiliations:** 1 Center for Biochemistry, Medical Faculty, University of Cologne, Joseph-Stelzmann-Str. 52, 50931 Cologne, Germany; 2 Center for Molecular Medicine Cologne (CMMC), University of Cologne, Robert-Koch-Strasse 21, 50931 Cologne, Germany

**Keywords:** Chronic inflammation, Cell biology

## Abstract

Necroptosis is a caspase-independent modality of cell death that requires the activation of the executioner MLKL. In the last ten years the field gained a substantial amount of evidence regarding its involvement in host response to pathogens, TNF-induced inflammatory diseases as well as pathogen recognition receptors (PRR)-induced inflammation. However, there are still a lot of questions that remain unanswered. While it is clear that there are specific events needed to drive MLKL activation, substantial differences between human and mouse MLKL not only highlight different evolutionary pressure, but also provide potential insights on alternative modalities of activation. While in TNF-induced necroptosis it is clear the involvement of the RIPK3 mediated phosphorylation, it still remains to be understood how certain inflammatory in vivo phenotypes are not equally rescued by either RIPK3 or MLKL loss. Moreover, the plethora of different reported phosphorylation events on MLKL, even in cells that do not express RIPK3, suggest indeed that there is more to MLKL than RIPK3-mediated activation, not only in the execution of necroptosis but perhaps in other inflammatory conditions that include IFN response. The recent discovery of MLKL ubiquitination has highlighted a new checkpoint in the regulation of MLKL activation and the somewhat conflicting evidence reported certainly require some untangling. In this review we will highlight the recent findings on MLKL activation and involvement to pathogen response with a specific focus on MLKL post-translational modifications, in particular ubiquitination. This review will highlight the outstanding main questions that have risen from the last ten years of research, trying at the same time to propose potential avenues of research.

## Facts

- Different plasma membrane-bound receptors and intracellular sensors can initiate signalling cascades culminating with MLKL activation and necroptosis.
- Upon RIPK3 phosphorylation, MLKL undergoes a conformation change that exposes its four helical bundle (4HB) domain, followed by membrane translocation and oligomerization.
- MLKL is specifically ubiquitinated during necroptosis following its phosphorylation.
- Ubiquitination regulates MLKL-mediated killing, either by promoting or repressing its cytotoxic potential.
- Ubiquitinated MLKL can fulfil cell death-independent functions, related to intracellular bacteria clearance.

## Open questions

- Can MLKL induce cell death in a RIPK3 independent manner?
- Does MLKL have any physiological role independent of RIPK3 and necroptosis?
- Why is MLKL controlled by IFN signalling and what is its biological significance?
- How can ubiquitination differentially regulate MLKL-killing potential?
- Do different cellular pools of MLKL exist, whose specific ubiquitination leads to distinct biological outcomes?
- Which is/are the E3 ligase(s) and DUB(s) that regulate the conjugation of ubiquitin to, and removal of ubiquitin from, MLKL?
- Does mono-ubiquitination vs poly-ubiquitination, and within the latter, the ubiquitin linkage type, differentially control MLKL activity?

## Introduction

Necroptosis is a caspase-independent form of programmed cell death whose most classical morphological features are cellular swelling and plasma membrane rupture [1, 2]. Between the end of the ’90 s and beginning of 2000s, in the quest to elucidate the role of caspases in cell death pathways driven by death receptors (DRs) such as TNFR1, Fas/CD95 and TRAIL-Rs, some groups reported a necrotic-like type of death that could be executed independent of caspases [3–5]. At the time, it was very intriguing the observation that inhibition of caspases, either via a synthetic compound (z-VAD) [6] or viral proteins (e.g., CrmA) [7], did not prevent DRs-induced cell death, but it rendered cells even more sensitive to death [3, 4]. Equally intriguing was the fact that this caspase-independent form of death needed the kinase activity of RIPK1 to be executed [5]. The discovery that Nec-1, a small molecule inhibitor, blocked this necrosis-like death by targeting RIPK1 was the ultimate proof of the existence of necroptosis as a novel, genetically encoded, form of cell death [8, 9]. The subsequent seminal discovery concerned the involvement of another kinase, RIPK3, in necroptosis [10–12]. RIPK3, via a RHIM-mediated homotypic interaction binds and interacts with RIPK1, leading to its autophosphorylation and consequent kinase activation, indispensable to trigger necroptosis [13–15]. Few years later, similar to the discovery of RIPK1, a novel necroptosis inhibitor, necrosulphonamide (NSA), was first identified and then linked to its target, MLKL, since then recognised to be the executioner of necroptosis [16]. This scientific journey, from the first evidence indicating the existence of programmed necrosis, till the discovery of MLKL [16, 17], lasted almost two decades. Instrumental in this journey were: i) the interrogation of death receptors (DRs) signalling pathways, in particular Fas and TNFR1, ii) the use of viral inhibitors of caspases, such as CrmA from Cowpox virus and B13R from Vaccinia virus [4] and iii) small molecule inhibitor screenings, that led to the identification of Nec-1 and NSA and finally, as always in science, the illimited curiosity and perseverance of scientists.

In this review we will summarize the latest advances on the understanding of the molecular mechanisms regulating MLKL activity. We will give particular emphasis to the role of the post-translational modifications, in particular ubiquitination, reported on MLKL and how they regulate its functions.

## MLKL structure and mechanisms of activation

**Fig. 1:**
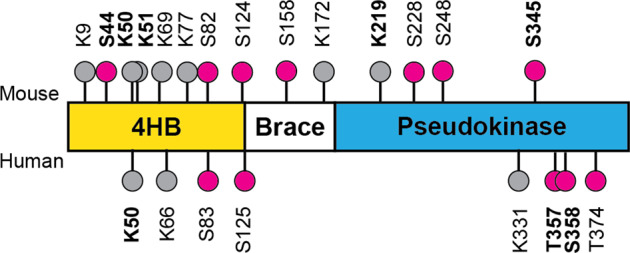
MLKL structure and functional reported post-translational modifications. MLKL domain composition and post-translational modifications. MLKL consists of a four helical bundle domain (4HB, aa 1–125), a brace region and a pseudokinase domain (aa 190–471 in human and 191–472 in mouse). MLKL undergoes RIPK3-mediated phosphorylation at threonine 357/serine 358 in human and serine 345 in mouse, shown by purple circles. In addition, MLKL can be ubiquitinated at different lysine residues, as shown by the grey circles. Sites in bold have been published to have a specific function.

The structure of MLKL in both human and mouse is characterised by two functional domains: the N-terminal four helical bundle domain (4HBD) and the pseudokinase domain (PSKD) [18] (Fig. 1). These two domains are connected by a brace domain consisting of two alpha helices [18, 19]. In both human and mouse, it has been shown that the 4HBD is absolutely required for MLKL to exert necroptosis. The forced expression of a dimerizable 4HBD in cells expressing MLKL leads to necroptosis in absence of any necroptotic stimulus or endogenous MLKL activation [20, 21]. This suggest that, under necroptotic stimulation, the post-translational modifications on MLKL lead to a structural active conformation that allows the 4HBD to be accessible and form oligo-multimeric structures with other active MLKL molecules, to execute necroptosis [22–25]. The PSKD can bind ATP without hydrolysing it, rendering this domain essentially inactive. However, this domain is necessary for RIPK3 binding and subsequent RIPK3-mediated MLKL phosphorylation: an event classified as the “molecular switch” [18, 25]. RIPK3-mediated phosphorylation is necessary to release the killer 4HBD in mouse, while in human it prompts MLKL interconversion to the close, but active form [26]. This in turn leads to i) the release of MLKL from RIPK3, ii) the formation of high order MLKL oligomers via 4HBD interaction, iii) translocation to the membrane culminating in pores formation, probably as a consequence of membrane destabilisation [18–24, 27–29]. It is clear that in both human and mouse the 4HBD is required for membrane permeabilization, although more efficiently in mouse than in human [26, 30].

In mouse, the phosphorylation event seems to be necessary to disrupt the hydrogen bond between the K219 and the Q343, required to keep MLKL in closed conformation. Mutation in any of these two residues (K219M or Q343A) disrupts the binding and allows MLKL active conformation, oligomerisation and killing [18, 31]. This would suggest that mouse MLKL does not require ATP for its activation. In human, MLKL has a closed-active-like conformation and a potential open-inactive conformation characterised by an equivalent K230-Q356 hydrogen bond [26]. Interestingly, under basal conditions, human MLKL has been reported to be bound to RIPK3 [26, 30] and, differently from mouse MLKL, the alanine substitution of K230 or Q356 is not sufficient to fully unleash MLKL activation and killing [16, 26]. Following a necroptosis stimulus, MLKL dissociates from RIPK3 as a consequence of RIPK3-mediated phosphorylation, which is now considered to be a destabilising event of the MLKL-RIPK3 interaction [26]. As a result of this dissociation, human MLKL acquires a closed- active conformation with the K230 bound to a E250 via a salt bridge, forming a so called intact catalytical C-spine. This, in turn, would require ATP or another metabolite ligand in the ATP- binding site to modulate the MLKL conformational change, toggling from active to inactive, and therefore impact on necroptotic kinetics [25, 26, 31].

Despite the similarities between the overall activation requirements, human MLKL and mouse MLKL are not interchangeable and mouse MLKL cannot complement the human necroptotic system and vice versa [21, 32, 33]. This has been justified specifically by the fact that mouse and human MLKL interact to, and are phosphorylated by their correspondent RIPK3. This suggests that, in different species, MLKL and RIPK3 have co-evolved in different ways [21]. To this day the phosphorylation of MLKL in its activation loop by RIPK3 is considered the most vital and essential post-translational modification required to execute necroptosis. While human MLKL is phosphorylated by RIPK3 on T357/S358, mouse MLKL is phosphorylated on the S345 [18, 20, 23, 29] (Fig. 1). However, it has been reported that in mouse, S124 in the 4HBD, S158 in the brace region as well as S228 and S248 in the PSKD are perhaps involved in the fine-tuning of MLKL killing activity [21]. In human, S125 was specifically identified to be phosphorylated following prolonged mitosis in HeLa cells (these cells do not express RIPK3) while the Y376 has been shown to be phosphorylated by TAM kinases, and contribute to MLKL stabilisation and MLKL oligomerisation [34, 35]. Recently, phosphomimetic substitution of different residues surrounding the activation loop of human MLKL showed that the T374D mutation completely inhibited necroptotic signalling by impairing RIPK3-mediated phosphorylation on T357/S358 [26]. Similar to S125, also T374 was identified to be phosphorylated in a cell cycle dependent-manner. The kinase(s) responsible for this phosphorylation events remain/s unknown. Recently, another study revelated that also the phosphorylation of S83 or S82, human and mouse MLKL, respectively, located in the 4HBD, inhibited necroptosis execution without impacting on  RIPK3-mediated MLKL phosphorylation [36]. Interestingly, while the mouse specific S345A (MLKL-phosphomutant) and S345D (MLKL-phosphomimic) mutations can completely inhibit or induce RIPK3 independent necroptosis, respectively, the human phosphomimic mutant (T357E/S358E) cannot activate necroptosis, neither in absence nor in presence of a stimulus [21, 23]. This seems to result from loss of binding between T357E/S358E-mutant MLKL and RIPK3, suggesting that human MLKL requires physical binding to RIPK3, in addition to phosphorylation, to exert its killing activity. The binding between human RIPK3 and human MLKL, in fact, seems to specifically require the formation of stable complexes between the RIPK3 kinase domain and MLKL PSKD. On the contrary, mouse RIPK3-MLKL interaction and consequent phosphorylation is based on the classical kinase ‘kiss and run’ observed and firstly identified in the RAS pathway [32, 33]. This is further supported by the fact that biochemically, differently from human, it is not possible to immunoprecipitate mouse RIPK3/MLKL complexes, suggesting perhaps a different stoichiometry as well as a different stability of the two species-specific complexes.

Following its activation MLKL has been reported to be ubiquitinated. This is described in detail below. Following these events MLKL molecules form oligomeric structures (trimers and/or multimers) of different size via 4HBD mediated interaction. It has been recently shown that this is an event that takes place in the cytoplasm and before membrane localisation. Once at the membrane, specific residues within the 4HBD bind phosphatidylinositol phosphate (PIPs) or cardiolipin [25, 27, 32, 33] This is required for human MLKL to travel from the cytosol to the plasma membrane, as well as other intracellular membranes, leading to plasma membrane recruitment, membrane destabilisation and cell death. While alanine substitutions of human D107 and E111 or mouse R63/D65, E102/K103, R105/D106, E109/E110 (all reported as double alanine mutations) completely abrogated necroptosis, alanine substitutions of the human K16/R17/K26/Q27/K50/R51 (five alanine mutations) inhibit plasma membrane recruitment and necroptotic cell death [19, 27, 33, 37]. Recently it has been shown that in mouse, K69, W108 and R107/Q138 (contained in the 4HBD) are specifically involved in lipid membranes permeabilization. Indeed, *Mlkl*^*−/−*^ cells reconstituted with the correspondent alanine single point mutations or double mutations, are protected from necroptosis [30].

The destabilisation of plasma membrane, the consequent pore formation and the precise composition of these pores in terms of active MLKL (phosphorylated or oligomerised) are currently not fully understood and a matter of intense investigation. The current size of the necroptotic pore is estimated to be 4 nm, however this does not seem to be sufficiently large to allow the release of some inflammatory molecules whose molecular size, in some case, is almost 100 KDa [38]. It is therefore possible that membrane tension of small pores might induce bigger ruptures in cells undergoing necroptosis. Moreover, the formation of a pore has never been shown to be physically/directly mediated by MLKL but rather by a membrane destabilisation that emerges as a consequence of the MLKL oligomers. Further studies, investigating the different stages of necroptosis in relation to pore sizes, MLKL activation status as well as MLKL localisation within the pore and membrane are clearly needed to fully answer these questions.

According to phosphosite.org, there are currently 43 reported post translational modifications in human MLKL, with the S358/T357 and the S125 being the most reported modification. Of these, only 29 are conserved, or potentially conserved, in mouse MLKL, however there is no evidence that such phosphorylation events are actually taking place in mouse. Most of these collated mass-spectrometry based proteomic experiments have been done in human cancer cell lines routinely used in many laboratories and in many cases the cellular system utilised does not even express RIPK3. This suggests that the reported phosphorylation sites might be markers for MLKL activation or function in cellular contexts that are not necroptosis dependent. It remains to be determined whether these post-translational modifications will show context dependent functions of MLKL that are currently not yet been identified and perhaps unveil MLKL involvement in cell signalling and/or cellular homeostasis independent of RIPK3. Of note, is the work unveiling the RIPK3-independent phosphorylation of mouse-MLKL on S44 (Fig. 1), that induces MLKL activation and consequent targeting of the myelin membranes. This, in turn, leads to the initial breakdown of the myelin sheaths following nerve injury [39].

## MLKL in TNFR1 signalling pathway

**Fig. 2:**
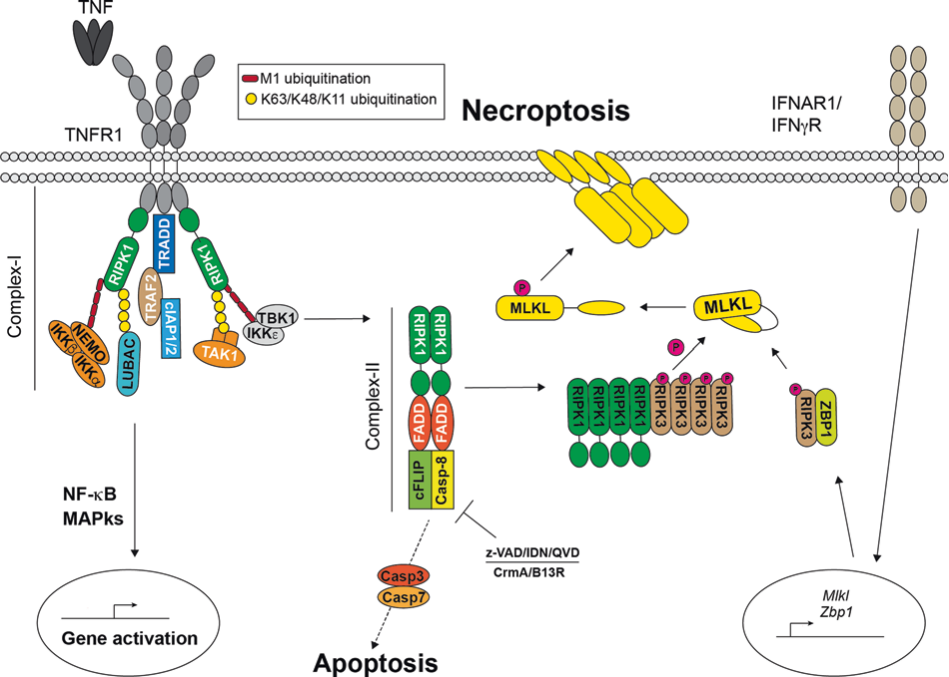
TNFIFN-induced necroptosis. Cartoon depicting TNFR1-induced signalling pathway and IFN mediated upregulation of MLKL and ZBP1, that can culminate with MLKL activation and necroptosis. Binding of TNF to TNFR1 triggers the formation of a membrane-bound complex referred to as complex-I. This complex is composed of adaptor proteins (e.g., TRADD and TRAF2), E3 ligases (e.g., cIAP1/2 and LUBAC) that synthesize poly-ubiquitin chains of different topology (i.e., K63, K48, K11 and M1) and kinases, such as RIPK1 and IKK1/2, and it leads to NF-kB and MAPKs activation and expression of pro-survival as well as pro-inflammatory genes. Alternatively, upon interferon receptors activation and/or IFN signaling activation, *Mlkl* and *Zbp1* are transcriptionally upregulated. Under certain circumstances, described in the main text, a secondary cytoplasmic complexes forms, referred to as complex-II. This complex promotes Caspase-8 activation and apoptosis. However, upon Caspase-8 inhibition by the means of synthetic or viral encoded caspase inhibitors, RIPK1 activates RIPK3 that in turn phosphorylates MLKL. Following interferon signaling, ZBP1 is upregulated and activated following binding to double-stranded RNA. Upon activation, ZBP1 binds to RIPK3 via RHIM/RHIM interaction, triggering RIPK3 phosphorylation and the consequent MLKL activation. Phosphorylated MLKL undergoes a conformation change to expose its 4 helical bundle (4HB) domain that promotes MLKL association with the plasma membrane. Here MLKL oligomerizes and disrupt plasma membrane integrity, causing necroptosis.

Necroptosis can be induced by a range of immune receptors, activated by their respective ligands [40]. Amongst the best characterized immune receptor/ligand systems in the context of necroptosis induction there is TNFR1/TNF [41] (Fig. 2).

TNF binding to TNFR1 triggers the formation of a membrane-bound complex referred to as complex-I [42], that is composed of adaptor proteins (e.g., TRADD and TRAF2), E3 ligases (e.g. cIAP1/2 and LUBAC) and kinases (e.g. RIPK1 and IKK1/2) [43–46]. Complex-I promotes the activation of the IKK1/2 kinases that in turn activate NF-κB via phosphorylation-induced degradation of IkBa [47]. However, under certain circumstances, such as cIAP1/2 depletion or genetic deletion of any of the LUBAC components (i.e., HOIP, HOIL-1 and SHARPIN) or inhibition of IKK activity, a secondary cytoplasmic complex forms, referred to as complex-II [45, 48–51]. This complex, composed of FADD, Capsase-8, cFLIP, RIPK1 and RIPK3, can induce apoptosis [42, 52, 53]. Upon Casaspe-8 inhibition, either pharmacological by a pan-caspase inhibitor (i.e., z-VAD-fmk, QVD-OPh or emricasan/IDN-6556), or virus-mediated (e.g., CrmA or B13R), or genetic (Caspase8 deletion or Caspase-8 catalytic inactivation), TNF induces RIPK1/RIPK3/MLKL-mediated necroptosis [3, 4, 54–59]. In fact, the embryonic lethality caused in mice by the deletion of Caspase-8 is prevented by the co-deletion of RIPK3 [54, 55].

Consistently with its role downstream of RIPK3, MLKL genetic deletion can rescue the embryonic lethality caused by Caspase-8 genetic deletion or Caspase-8 inactivating mutation [58–60]. Interestingly, activation of necroptosis can also happen in absence of RIPK1 [61, 62]. This was an important discovery that defined the role of RIPK1 as a homeostatic regulator of cell death and, importantly, as an inhibitor of both necroptosis and apoptosis in vivo. Accordingly, loss of RIPK1 drives TRADD recruitment to FADD via DED domain interaction, leading to activation of Caspase-8, and RHIM-mediated ZBP1 interaction with RIPK3 and activation of MLKL [63–65] (Fig. 2). Co-deletion of either RIPK3 or MLKL does not rescue the lethality of the RIPK1 deficient mice, due to the activation of aberrant apoptosis via Caspase-8 [61, 62].

Further investigation revealed that MLKL drives a necroptosis-dependent harmful phenotype in a number of tissue-specific, genetically modified mice, such as *RIPK1*^*E-KO*^ (epidermal RIPK1 deletion) [66], *Caspase-8*^*C362S/E-KO*^ (skin specific Caspase-8 inactivation) [58] and *Fadd*^*IEC-KO*^ (intestinal epithelial cell-specific deletion of *Fadd*) [67]. Of note is the fact that, in some instances, the deletion of RIPK3 and MLKL do not phenocopy each other. The most sticking example being the different effect of *Ripk3* vs *Mlkl* deletion in the *Sharpin*^*cpdm/cpdm*^*Caspase-8*^*−/−*^ background. Indeed, while *Sharpin*^*cpdm/cpdm*^*Caspase-8*^*−/−*^*Ripk3*^*−/−*^ mice are not viable, *Sharpin*^*cpdm/cpdm*^*Caspase-8*^*−/−*^*Mlkl*^*−/−*^ mice are healthy [51]. Similarly, while the *Caspase-8*^*C362S/C362S*^*Ripk3*^*−/−*^ mice are viable, the *Caspase-8*^*C362S/C362S*^*Mlkl*^*−/−*^ die perinatally [58, 59]. This underscores the fact that RIPK3 has necroptosis-independent functions, possibly involved in survival, which in the case of the *cpdm* mutation are unmasked in the context of attenuated linear chains formation. At the same time this also opens the possibility that, again, in the context of attenuated linear chains, MLKL killing potential could be unleashed in a RIPK3 independent manner, via a mechanism that is still completely unknown.

## MLKL in TLRs signalling pathway

**Fig. 3:**
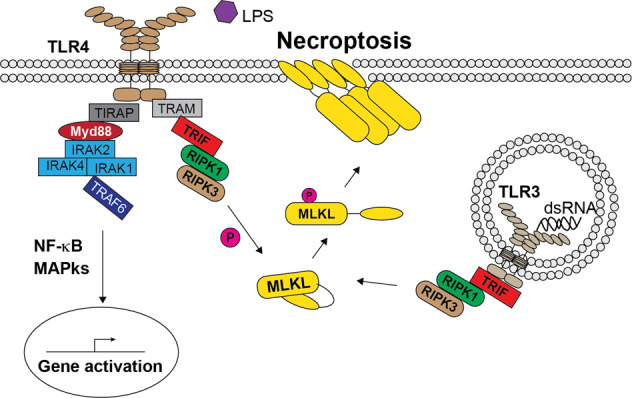
Toll like receptors (TLRs)-induced necroptosis. Schematic representation of how TLR3 and TLR4 can induce MLKL activation and necroptosis. TLR4 activation by LPS triggers the formation of two different signalling complexes. One, name the Myddosome, composed of the adaptor protein TIRAP, Myd88, IRAKs and TRAF6, activates NF-κB and MAPKs for the expression of pro-inflammatory genes. The other, referred to as Triffosome, is composed of the adaptor protein TRAM and TRIF. TRIF, via its RHIM domain, can recruit RIPK1, depending on the cell type, and RIPK3. RIPK3 in turn activates MLKL via phosphorylation. Activated MLKL will in turn execute necroptosis. Activation of TLR3, that localizes at endosomal membranes, by dsRNA determines the recruitment of TRIF that in turn recruits RIPK1, depending on the cell types, and RIPK3. RIPK3 phosphorylates MLKL causing its activation, translocation to the membrane, oligomerization and, ultimately, necroptosis.

Other immune receptors apart from TNFR1 have the ability to induce necroptosis [40]. Toll-like receptors (TLRs) are pattern recognition receptors (PRR), able to sense highly conserved pathogen-derived molecules or molecules released by damage cells [68, 69]. In particular, TLR3 and TLR4 have been shown to induce necroptosis independent of TNF [70] (Fig. 3). TLR3 is localized in the endosomal compartment and is activated by dsRNA, a viral replication intermediate [71]. After activation, the adapter molecule TRIF is recruited to TLR3. TRIF can in turn recruit TBK1, via the pLxIS motif, for the activation of the Type I interferon pathway [72], and RIPK3 via the RHIM domain [73]. In conditions of Caspase-8 inhibition, TLR3 stimulation induces MLKL-dependent necroptosis [74]. Of note, while in macrophages both RIPK1 and RIPK3 are needed for MLKL activation, in fibroblast and endothelial cells RIPK1 is dispensable [74]. However, at present it is still not known how, in the absence of RIPK1, Caspase-8 could get into close proximity with RIPK3, since RIPK3 has neither a DD nor a DED domain. Conversely, RIPK1, via its DD-mediated interaction with FADD, can recruit Caspase-8 to the complex.

TLR4, upon binding to LPS, dimerizes and the dimerized receptor is bound by the adapter protein TIRAP. This triggers the formation of a complex referred to as Myddosome, where Myd88 is required to promote NF-κB signalling via the recruitment of TRAF6 [75]. In addition, TLR4 can also recruit TRIF and therefore RIPK kinases [70, 74]. Similar to the case of TLR3, RIPK1 is needed for MLKL-dependent necroptosis triggered by LPS in combination with Capsase-8 inhibition in macrophages, while it is dispensable in fibroblast and endothelial cells. Furthermore, when cIAPs depletion and XIAP inhibition were combined to LPS stimulation in the presence of Caspase-8 inhibition, macrophages undergo a RIPK1-independent, RIPK3-dependent activation of MLKL. This resulted in NLRP3 inflammasome activation and IL-1β production, most probably as a consequence of MLKL-mediated membrane damage occurring during necroptosis [76–78]. Therefore, different types of immune signalling pathway can converge into the activation of MLKL, provided Caspase-8 activity is compromised. MLKL-mediated membrane damage and necroptosis do not only kill the cells, but can trigger further events, such as inflammasome activation [79, 80], that might have relevant pathophysiological consequence on bystander cells.

## MLKL in viral infection

Necroptosis is believed to have originally evolved among different species as an innate immune response against pathogens and their ability to inhibit apoptosis [81, 82]. As a consequence, the ability of host cells to activate necroptotic cell death and prevent viral replication has contributed to additional evolutionary pressure on pathogens, leading to the development of (pathogen)-specific mechanisms to avoid necroptosis [83]. In a time-dependent manner, many viruses can counteract necroptosis to efficiently hijack the cellular machinery required for viral replication [81]. Once replicated, viruses can either induce necroptosis to take advantage of the MLKL-induced cellular burst to exit the cells or keep necroptosis under control and localise at the host outer plasma membrane to form exosomes-like structures and exit via an exosome-mediated mechanism [82]. In some cases, virus that can inhibit necroptosis in human would conversely induce it in mouse. This highlights the incredible evolutionary pressure, driven not only by pathogens but also by hosts, that both MLKL and RIPK3 have undergone. Involvement of MLKL in viral infection/response can be divided into two categories: RIPK3 mediated activation of MLKL (Vaccinia virus, Cytomegalovirus, Influenza virus) and MLKL direct targeting (BeAn 58058 poxivirus (BAV), Cotia Poxvirus (COTV) and human HCMV UL36) [84, 85]. It is currently unknown why some viruses would target one over the other or even both. It is evident in the literature that the majority of the studies, looking at the role of necroptosis in viral infection, have focused on RIPK3 and its activation. Many studies have solely focused on RIPK3 due to the absence of a MLKL knock-out mouse model (at the time) or simply of the knowledge of MLKL as an executioner of necroptosis. Differently from *Ripk3, Mlkl* mRNA is usually increased during inflammation, especially as a consequence of interferon type I and II signalling (mostly occurring during pathogen infection [86] (Fig. 2). This has been shown in several reports and following direct treatments with IFNγ and/or LPS [87]. Moreover, activation of the transcription factors STAT1, STAT2 and IFN regulatory factor 9 (IRF9) via IFNAR or IFN-type I signalling, as well as cGAS/STING activation of STAT1, have been shown to increase MLKL levels both transcriptionally and consequently translationally [28]. On the other hand, *Ripk3* mRNA (and not MLKL) is upregulated after certain infections (such as: *Mycobacterium Tubercolosis* and *Clostridium difficile*) [86], independent of interferon signalling, via mechanisms that involve promoter demethylation [88]. Given the differential transcriptional upregulation of MLKL and RIPK3, one could hypothesise that these two molecules might also have alternative roles in the response to viral infection or inflammation, independent from each other. Alternatively, this might also suggest, independently thereof, that a specific threshold of MLKL expression must be reached to unleash canonical necroptosis while RIPK3 expression can remain constant to reach a specific stochiometric proportion. Below we summarise our current understanding of necroptosis involvement in viral infection.

**Fig. 4:**
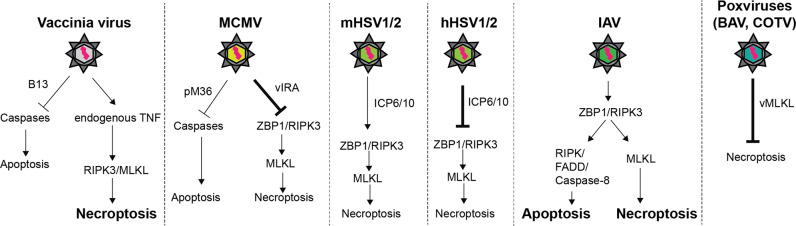
MLKL-mediated necroptosis in viral responses. Schematic representation of the involvement of MLKL-mediated necroptosis in host anti-viral responses. While Vaccinia virus, mouse herpes simplex virus (mHSV) and Influenza virus (IAV) induce the activation of necroptosis, mouse cytomegalovirus (MCMV), human herpes simplex virus (human HSV) and Poxviruses such as BAV and COTV have evolved strategies to block MLKL-induced necroptosis.

The most known example is the Vaccinia virus (VV). During infection VV induces TNF production, driving RIPK3 activation and consequent MLKL induced necroptosis. This is a host-induced protective mechanism, in fact RIPK3 deficient mice succumb to VV infection while WT mice can protect themselves mounting an immune response triggered by necroptosis induced inflammation [4] (Fig. 4). Different from VV; mouse Cytomegaloviruses, BeAn 58058 poxivirus (BAV), Cotia Poxvirus and human Herpes simplex virus 1 and 2 (HSV1/2) have evolved mechanisms to inhibit necroptosis induction [82, 84, 85, 89–93] (Fig. 4).

Mouse Cytomegalovirus (MCMV) gene m45 expresses the viral inhibitor of RIP activation (vIRA) which targets the RHIM domain of RIPK3 to suppress necroptosis by inhibiting RIPK3 binding to ZBP1 [82, 89]. This activation axis is known to be specifically engaged in mouse CMV (MCMV) infected cells and specifically inhibited by vIRA, that indirectly antagonises MLKL activation and execution of necroptosis [89, 94] (Fig. 4). In the case of HSV1-HSV-2, the ICP6 and ICP10 proteins, respectively, can bind RIPK3 in a RHIM dependent manner [92, 95]. While in mouse these lead to activation of necroptosis, in human their RHIM-RHIM interaction with RIPK1 or RIPK3 leads to disruption of necrosome and inhibition of necroptosis [91, 95] (Fig. 4).

Influenza A virus (IAV), instead, seems to induce RIPK3 accumulation by inhibiting cIAP2 [96]. Interestingly, neither virus infectivity nor replication is impeded in the absence of RIPK3. Following infection, direct activation of RIPK3 mediates recruitment of FADD, RIPK1 and MLKL to drive both MLKL induced necroptosis and RIPK3/Caspase-8 mediated apoptosis [97]. RIPK3, in fact, seems to nucleate RIPK1 and FADD leading to Caspase-8 recruitment and consequent apoptosis (Fig. 4). Cells deprived of MLKL expression die of pure caspase-dependent cell death while RIPK3 deficient cells present a significant inhibition of both apoptosis and necroptosis.

Recent work has shown that the DNA sensor ZBP1/DAI is responsible for nucleation of RIPK3, necrosome formation (Fig. 2) and downstream activation of both MLKL and Caspase-8 in IAV-infected cells [98]. Additionally, ZBP1 can trigger pyroptosis following IAV infection. This indicates that ZBP1 can act as the apical sensor for IAV infection. Consequently, cells lacking ZBP1 are much more resistant to virus-induced cell death than the RIPK3-deficient cells. Indeed, while ZBP1/RIPK3 interaction nucleates the necrosome to drive MLKL activation, in absence of RIPK3, ZBP1 can bind directly to RIPK1 and drive RIPK3-independent, FADD/RIPK1/Caspase-8-dependent apoptosis. Loss of RIPK3 or ZBP1 or the combined loss of either with a non-cleavable Caspase-8 would drive unsustainable IAV replication leading to lethal infection, compromising host viral defense and as a consequence of apoptosis and necroptosis inhibition [97, 99]. Interestingly, loss of MLKL alone does not lead to any significant difference in susceptibility to IAV infection, probably due to the residual apoptosis still exerted by the ZBP1/Caspase-8 axis. Indeed, combined loss of RIPK3 and FADD renders mice susceptible to IAV-induced lethality, due to the incapability of the infected cells to undergo cell death and evoke an anti-viral response [100].

Recently, few reports have emerged showing a direct targeting of MLKL and consequent inhibition of necroptosis. Poxviruses such as BeAn 58058 poxvirus (BAV) and Cotia poxvirus (COTV), express truncated MLKL viral homologues. These “viral MLKL” forms only possess a PSKD lacking the N-terminal 4HBD domain. They function as inhibitors of necroptosis by replacing host MLKL as the target of RIPK3-mediated phosphorylation. Consequently, host MLKL remains inactive, and necroptosis cannot be executed [85] (Fig. 4).

Interestingly, human CMV, differently from the mouse CMV, inhibits necroptosis not via the expression of a vIRA like protein. The expression of an early-regulated element, IE1, prevents cell death at a stage that follows MLKL phosphorylation, preventing membrane leakage [101]. The authors of the papers discuss the possibility that this element changes the cellular environment that allows necroptosis execution, however, it is not fully understood how and what mechanism could be responsible for such outcome. Recently, it has been shown that the product of human CMV UL36 binds both mouse and human MLKL, however, it only drives the degradation of human MLKL, blocking necroptosis execution in human infected cells [84]. The ability of viruses to directly target MLKL is rather interesting, considering their established strategies to modulate RIP kinases. What pressure would induce viruses to develop targeting mechanisms also against MLKL? Maybe this is an indication of other unknown functions of MLKL in viral infection which are yet to be discovered, or maybe the ability of MLKL to feed back into other modalities of cell death similarly to RIPK3 in the case of influenza infection. It will be interesting to determine if other viruses including Sars-CoV-2 and its variants can also encode for such MLKL-like decoy-substrates and perhaps investigate if MLKL is required for other homeostatic responses following viral infection rather than merely cell death.

## MLKL ubiquitination

The ubiquitin system plays a relevant role in necroptotic signalling regulation [102]. Several different lysine residues on RIPK1 have been demonstrated to be ubiquitin acceptor. Intriguingly, RIPK1 ubiquitination can both promote and prevent necroptosis. Indeed, while for example K376 [103–105] or K634 [103] ubiquitination restrains RIPK1 kinase activity and necroptosis, ubiquitin conjugation at lysine 115 [106] or 627 [107] promotes necroptosis. RIPK3 has also been described to undergo ubiquitin-mediated regulation during necroptosis. The K5 residue ubiquitination promotes the ability of RIPK3 to induce necroptosis and the ubiquitination status of this residue is directly controlled by the deubiquitinase A20 [108]. MLKL was also reported to undergo ubiquitination in the context of LPS signalling pathway [77], however, whether or not ubiquitin could control MLKL killing potential was elucidated only recently. Between 2021 and early 2022 [109–111], three reports were published, deeply dissecting the role of ubiquitination in MLKL biology. Each of these three reports attributes to MLKL ubiquitination a different biological role, and, although results are to some extent in conflict, they offer highly interesting perspectives on MLKL regulation that will be matter of investigation in the coming years. The different conclusions reached in these three studies could be due, at least in part, to the different ubiquitination sites that were identified and characterized. Species- (human vs mouse) and localization- (membranes vs cytoplasm) related reasons might also explain the different findings obtained in the three reports.

**Fig. 5:**
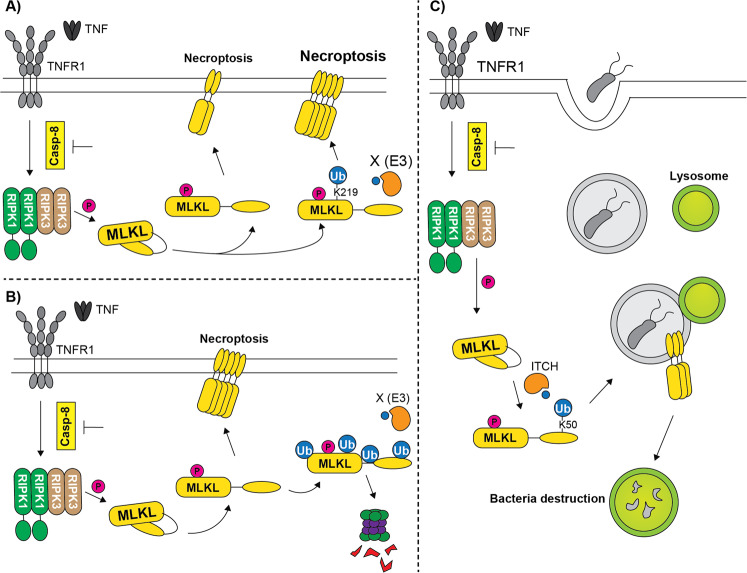
Proposed roles for ubiquitin modifications on MLKL. **A** TNFR1 stimulation, in the presence of caspase inhibition, promotes MLKL ubiquitination at K219, by a so far unknown E3 ligase. This ubiquitination events contributes to MLKL ability to oligomerize and induce necroptosis. **B** TNFR1 activation and caspase inhibition trigger MLKL multi-mono-ubiquitination, that represents a signal for MLKL degradation via the proteasome. The E3 ligase involved in this process is still unknown. **C** TNFR1 activation, concomitantly with caspase inhibition, stimulates ubiquitination of MLKL at K50. Ubiquitin-modified MLKL enhances then lysosome-mediated destruction of intracellular bacteria.

The first report, in chronological order, addressing the role of MLKL ubiquitination is from the group of P. Meier [111]. Here the authors unmistakably showed that MLKL undergoes ubiquitination in a time-dependent manner during necroptosis and the earliest ubiquitin modifications coincide with MLKL phosphorylation and the onset of necroptosis. This ubiquitin modifications require RIPK1 and RIPK3 kinase activity since they can be prevented by RIPK1 and RIPK3 kinases specific inhibitors. This ubiquitination mainly occurs prior to the MLKL oligomerization and translocation to the membrane, as shown using PLA (proximity ligation assay) and subcellular fractionation. Furthermore, the authors showed that the main ubiquitin linkage type present on MLKL during necroptosis is K63. In order to strengthen the correlation between MLKL ubiquitination and necroptosis, the authors perform a di-Gy immunoprecipitation in lysates of cells undergoing necroptosis that was followed by mass spectrometry analysis. This led to the identification of 4 ubiquitin-acceptor lysine residues, K51, K77, K172 and K219 (Fig. 1). By using mouse *Mlkl*^*−/−*^ cell reconstitutions with single or multiple lysine-mutant versions of MLKL, the authors concluded that the K219 residue (conserved in human, K230) played a particularly prominent role in MLKL-killing potential. Of note, the K219R mutant displayed significantly less ubiquitination than the WT MLKL during necroptosis. Accordingly, MDFs and BMDMs isolated from a newly generated mutant mouse, the *Mlkl*^*K219R*^, were resistant to TNF-induced necroptosis. Similarly, the *Mlkl*^*K219R*^ mouse was resistant to necroptosis-mediated skin damage. K219 is a residue important to form a hydrogen bond with Q34,3 that keeps MLKL in an inactive state. Phosphorylation at S345 mediates a conformational change that destabilizes this hydrogen bond and triggers MLKL activation. The model the authors proposed indicates that following phosphorylation-mediated activation of MLKL, the K219 becomes available to accept ubiquitin, therefore stabilizing the active conformation of MLKL and contributing to its killing potential (Fig. 5A).

Later in the same year, the group of J. Silke also published a study where they investigated the function of MLKL ubiquitination in necroptosis, coming to opposing conclusions [109]. Indeed, the authors of this study provided evidence supporting a model whereby ubiquitination of MLKL antagonizes necroptosis. Of note, they confirmed what found in the previous study about the fact that (i) MLKL is ubiquitinated during necroptosis, (ii) RIPK1 and RIPK3 kinase activity are required, both in mouse and human, (iii) MLKL is ubiquitinated at multiple sites, however, they did not detect ubiquitination on the K219 and iv) ubiquitinated MLKL is localized at the plasmamembrane. Conversely, they indicate that (i) the ubiquitination occurs in the crude membrane fraction and not in the cytoplasm and follows MLKL oligomerization, and (ii) MLKL only undergoes multi-mono-ubiquitination, rather than poly-K63 ubiquitination. By making use of a N-terminal Flag-MLKL, that can undergo phosphorylation, ubiquitination and translocate to the membrane but not permeabilize the membrane and kill cells, they observed that after an initial membrane accumulation, it diminished over time. This time-dependent decrease could be delayed by lysosomal and proteasomal inhibitors, indicating that indeed ubiquitination favours MLKL degradation via either the lysosomes or the proteasome or both. This, as the authors suggested, might represent a mechanisms cells employ to restrain MLKL cytotoxic potential and somehow control the extent of necroptosis once it is activated. In addition, the authors, by fusing MLKL to the deubiquitinase USP21 [112] that removes ubiquitin modifications from MLKL, showed that MLKL deubiquitination can induce cell death, although to a minor extent, independent of necroptosis stimulation. This would suggest that a fraction of MLKL constantly undergoes plasma membrane translocation where it is turned over in a ubiquitination-dependent manner (Fig. 5B). This last might be a mechanism for the cells to always have their guard up in case of pathogen attack and to be able to quickly unleash MLKL killing power.

A third group, led by D. Wallach, also investigated the biological function of MLKL ubiquitination, coming to a yet different conclusion [110]. In this study, they attributed a cell death-independent function to MLKL ubiquitination. Similar to the other two studies, their initial observation was that MLKL undergoes ubiquitination following necroptosis-inducing treatments, both in human and mouse cells. These ubiquitin modifications required MLKL phosphorylation and oligomerization to occur and where manly of the K63 linkage type. They also identified by mass spectrometry several ubiquitin-modified lysine residues, with K50 (human) and K50/K51 (mouse) accounting for most of the ubiquitin modifications (Fig. 1). However, given the small effect on MLKL cytotoxic potential generated by mutating these lysine residues, the authors concluded that ubiquitin modifications on MLKL might serve a function other than necroptosis. The authors had the intriguing observation that ubiquitinated MLKL was found almost exclusively in the microsomal fraction and, in particular, it co-localized with endosomal membranes, both early and late endosomes, upon necroptotic stimulation. Interestingly, it was the ubiquitination mediated by a specific E3 ligase, ITCH, that determined this specific subcellular localization of MLKL. Indeed, the K50R mutant was not found in the endosomes. The ability of MLKL to localize into the endosome in a ubiquitin-dependent manner during activation of canonical necroptosis correlated with the ability of cells to clear intracellular bacteria, such as *Listeria Monocytogenes* and *Yersinia Enterocolitica*. It was previously reported that MLKL can mediate intracellular bacteria clearance independent of its ability to trigger necroptosis, by binding directly to the pathogens in the cytosol [113]. Here, the authors provided further insights regarding the anti-bacterial functions of MLKL. In fact, they showed that ubiquitinated MLKL mediates the killing of bacteria when they are still confined to the membrane fraction, before their translocation to the cytosol. Accordingly, cell reconstituted with the K50R mutant that cannot be ubiquitinated and cannot bind endosomes, cannot clear bacteria as efficiently as WT MLKL reconstituted cells. Of note, the K50R mutant MLKL can still bind and clear the cytosolic bacteria. Furthermore, K50R mutant cells infected with a *L. Monocytogenes* strain that cannot translocate to the cytoplasm from the membrane fraction completely lose the ability to prevent bacteria replication. The model proposed is consistent with MLKL undergoing ubiquitination during necroptosis. This probes MLKL to associate with endosomes and promote the disposal of intracellular bacteria by favouring their endosomal trafficking and lysosomal destruction. Importantly, this occurs before MLKL triggers plasma membrane disruption and necroptosis (Fig. 5C). From an evolutionary point of view, ubiquitination of MLKL might represent a mechanism that contributes to the clearance of intracellular bacteria before plasma membrane rupture, to prevent the release of live pathogens by bursting cells.

It is very intriguing to remark how these three different groups have come up with three different models regarding how ubiquitination controls MLKL functions. While according to one group ubiquitination promotes MLKL-mediated killing, another group claims the exact opposite. A third group, proposes that MLKL ubiquitination serves yet another purpose, to help bacterial clearance in a cell death-independent manner. Different hypothesis can be made to explain the observed differences, at least with regard to cell death-related role of MLKL ubiquitination. For example, the fact that only one of the three groups identified by mass spectrometry K219 as a ubiquitin acceptor and that this K219 is the only residue whose mutation modulates MLKL activity among all other identified lysine residues, allowed that same group to conclude on the pro-killing role of MLKL ubiquitination. Conversely, the other two groups, that did not identify K219 and could not correlate a specific ubiquitination event to cell death induction, used other strategies/systems to attribute a function to MLKL ubiquitination. The fact that lysine residue mutagenesis employed by the Silke and Wallach groups did not allow to establish a correlation between a particular lysine modification and necroptosis modulation is not entirely surprising. Indeed, it is often the case that in absence of a ubiquitination site the promiscuity of this post-translational modification might target an alternative site. Since the K219 modification was not identified by two of the three groups, the K219 ubiquitination might indeed represent a small fraction of the entire pull of ubiquitinated MLKL. Still, this small pool of MLKL modified at K219, has important necroptosis modulatory functions.

The group of J. Silke reached the conclusion that ubiquitination is a signal for degradation by using a FLAG-tagged version of MLKL as well as a USP21 fusion construct. Although FLAG-MLKL undergoes the same modifications and activation steps as WT MLKL, it is not capable of killing cells. Therefore, one can postulate that if MLKL accumulates at the membrane in its oligomeric forms, but necroptosis can’t be executed, cells have in place ubiquitin dependent mechanisms to turn MLKL over. This could even imply that MLKL undergoes ubiquitination events both in the cytoplasm and at the plasma membrane and that they differ in chain specific-type and function (i.e., pro cell death in the cytoplasm and anti-cell death at the membrane). Hence, different pools of MLKL, localised differently in the cell, might be ubiquitinated differently to further regulate the extent of necroptosis in a time- and place-specific manner. The USP21 fusion construct, however, would suggest that overall, ubiquitination, independent of its quality, inhibits necroptosis execution. Therefore, upon a strong pathophysiological necroptotic stimulus, ubiquitinated MLKL rapidly induces necroptosis and ubiquitination at K219 helps to maximise the MLKL oligomerization and killing. Conversely, in conditions where the strength of the necroptotic stimulus is sub-optimal, MLKL still translocates to the membrane, but it does not exceed the threshold required for the killing, maybe as a consequence of lack of K219 ubiquitination. In these settings MLKL, stuck the membrane, might undergo further ubiquitination that causes its degradation.

## Concluding remarks

It is clear that MLKL, in a species-dependent manner, is under a tight regulatory control. RIPK3-mediated phosphorylation plays an important role in its initial activation and is clearly the first checkpoint required to successfully engage necroptosis. While it remains unclear if and how MLKL can truly drive cell death independently of RIPK3, it is unlikely that TNF-induced necroptosis might take place via the phosphorylation mediated by another kinase on a different site from the S345 and S357/T358. It is however possible that MLKL might be involved in different signalling events as already shown, or even alternative caspase-independent modalities of cell death that would potentially activate MLKL via currently unknown mechanisms, similarly to nerve injury. Recently a second checkpoint has emerged that needs to be satisfied to properly control MLKL killing potential. Published studies have highlighted the importance of ubiquitin modifications in modulating the extent of necroptosis.

Under which different pathophysiological conditions this might happen and which would be the E3 ligases involved in the ubiquitination of different pools of MLKL still needs to be understood and will require further efforts from researchers. What we learnt from these three different studies is that MLKL is ubiquitinated during necroptosis and that ubiquitination represents an important regulatory checkpoint for MLKL activity in addition to phosphorylation. Understanding the ubiquitin-mediated regulation of MLKL will be highly relevant in contexts where necroptosis plays a pathophysiological role, such as viral infections, autoinflammatory syndromes and tumour immunity. Finally, and very intriguingly, the investigation of MLKL ubiquitination opened up new perspectives regarding potential necroptosis-independent roles of MLKL. Altogether, the reported post-translational modifications, whose function is known or still unknown, undoubtedly point at a complex level of regulation which, in a context dependent manner, will certainly place MLKL as an important executioner of different biological outcomes yet to be discovered.
